# Differential diagnosis between chronic pancreatitis and pancreatic cancer: value of the detection of *KRAS2* mutations in circulating DNA

**DOI:** 10.1038/sj.bjc.6600475

**Published:** 2002-08-27

**Authors:** F Maire, S Micard, P Hammel, H Voitot, P Lévy, P-H Cugnenc, P Ruszniewski, P Laurent Puig

**Affiliations:** Fédération Médico-Chirurgicale d'Hépato-Gastroentérologie, Hôpital Beaujon, AP-HP, 92110 Clichy, France; U490 INSERM Laboratoire de Toxicologie Moléculaire, 45 rue des Saints-Pères 75006 Paris, France; Laboratoire de Biochimie, Hôpital Beaujon, 92110 Clichy, France; Service de Chirurgie Digestive et Oncologie, Hôpital Européen Georges Pompidou, 75015 Paris, France

**Keywords:** *KRAS2* mutations, circulating DNA, pancreatic adenocarcinoma, chronic pancreatitis

## Abstract

*KRAS2* mutations in codon 12 have been detected in about 80% of pancreatic cancers. The aim of this study was to evaluate the value of *KRAS2* mutations detection in circulating deoxyribo nucleic acid to differentiate pancreatic cancer from chronic pancreatitis. Circulating deoxyribo nucleic acid was isolated from serum in 47 patients with histologically proven pancreatic adenocarcinomas (26 males, median age 65 years) and 31 controls with chronic pancreatitis (26 males, median age 48 years). Mutations at codon 12 of *KRAS2* gene were searched for using polymerase chain reaction and allele specific amplification. Serum carbohydrate antigen 19.9 levels were also determined. *KRAS2* mutations were found in 22 patients (47%) with pancreatic cancer and in four controls with chronic pancreatitis (13%) (*P*<0.002). None of the latter developed a pancreatic cancer within the 36 months of median follow-up. The sensitivity, specificity, positive and negative predictive values of serum serum *KRAS2* mutations for the diagnosis of pancreatic cancer were 47, 87, 85 and 52%, respectively. *KRAS2* mutations were not related to age, gender, smoking habit, tumour stage, or survival. Among the 26 patients with normal or non-contributive (due to cholestasis) serum carbohydrate antigen 19.9 levels, 14 (54%) had *KRAS2* mutations. The combination of *KRAS2* and carbohydrate antigen 19.9 gave a sensitivity, specificity, positive and negative predictive values for the diagnosis of pancreatic cancer of 98, 77, 87 and 96%, respectively. Detection of *KRAS2* mutations in circulating deoxyribo nucleic acid has a low sensitivity but a specificity about 90% for the diagnosis of pancreatic cancer. It seems particularly useful when serum carbohydrate antigen 19.9 levels are normal or inconclusive. A combined normal serum carbohydrate antigen 19.9 and absence of circulating *KRAS2* mutations makes the diagnosis of pancreatic cancer extremely unlikely.

*British Journal of Cancer* (2002) **87**, 551–554. doi:10.1038/sj.bjc.6600475 www.bjcancer.com

© 2002 Cancer Research UK

## 

Five-year survival in patients with pancreatic adenocarcinomas is less than 5%, partly due to advanced disease at diagnosis. The differentiation between pancreatic cancer and chronic pancreatitis can be particularly difficult leading to inappropriate treatment. Serum carbohydrate antigen 19.9 (Ca 19.9) levels are elevated in 80% of pancreatic cancer patients, but can also be increased in 20% of patients with chronic pancreatitis (Satake and Takeuchi, 1994; Nouts *et al*, 1998). Moreover, pancreatic inflammation, as observed in chronic pancreatitis, can be mistaken on imaging as cancer and inversely. An accurate and non-invasive test to differentiate pancreatic cancer from chronic pancreatitis would be extremely helpful.

Previous studies have reported *KRAS2* gene mutations (almost always confined to codon 12) in 75 to 95% of exocrine pancreatic cancer (Caldas and Kern, 1995). *KRAS2* mutations provoke activation of nuclear transcriptor factors, resulting in cellular proliferation and also in tumour angiogenesis as reported recently (Banerjee *et al*, 2000; Ikeda *et al*, 2001). Detection of *KRAS2* mutations were first reported in surgically removed pancreatic tumoural tissue or at autopsy (Almoguera *et al*, 1988; Tada *et al*, 1991). Thereafter mutations were discovered in 63 to 83% of samples of pure pancreatic juice or main pancreatic duct brushing obtained during endoscopic retrograde pancreatography (Iguchi *et al*, 1996; Kondo *et al*, 1997; Tada *et al*, 1998; van Laethem *et al*, 1998; Okai *et al*, 1999; Watanabe *et al*, 1999; Ha *et al*, 2001; Pugliese *et al*, 2001; Seki *et al*, 2001) or at fine-needle tumour aspiration (Pabst *et al*, 1999; Puig *et al*, 2000), and in 20 to 54% of stools (Caldas *et al*, 1994; Wenger *et al*, 1999) from patients with pancreatic cancer. Circulating deoxyribo nucleic acid (DNA) was first detected in serum or plasma of normal subjects in 1975 (Steinman, 1975). Since then, mutations in the *KRAS2* gene have been detected in the plasma of patients with colorectal, lung and haematological cancers (Anker *et al*, 1997). To date, few studies have reported *KRAS2* mutations in circulating DNA in patients with pancreatic cancer with a wide spectrum of sensitivity (27 to 81%) (Sorenson *et al*, 1994; Mulcahy *et al*, 1998; Yamada *et al*, 1998; Castells *et al*, 1999; Porta *et al*, 1999; Theodor *et al*, 2000). The aim of our study was to evaluate the value of *KRAS2* mutation detection in circulating DNA in a large series of patients to differentiate pancreatic adenocarcinoma from chronic pancreatitis.

## PATIENTS AND METHODS

### Selection and outcome of patients

Between January 1995 and 1999, 47 patients (26 males and 21 females, median age 65 years (range 39–84)) with pancreatic ductal adenocarcinoma were included in the study. In all of them, diagnosis was confirmed by pathological examination of pancreatic tumour obtained by fine needle aspiration during endoscopic ultrasonography (*n*=42) or operative procedure (*n*=5). Tumour staging was established by abdominal computed tomography, endoscopic ultrasonography or operative findings: stage I (*n*=5, 11%), stage II or III (*n*=19, 40%) and stage IV (*n*=23, 49%) according to the TNM classification (Uicc, 1997). Five patients underwent surgical resection, 32 patients received systemic chemotherapy and/or radiotherapy and 10 symptomatic treatment. Median follow-up was 6 months (range 1–24). At the end of the study, all but four patients with pancreatic cancer were dead.

### Control group

A control group was recruited during the same time in the same centre and included 31 patients with chronic pancreatitis (26 men and five women, median age 48 years (range 20–64)). Diagnosis of chronic pancreatitis relied upon the presence of pancreatic calcifications and/or irregularity of pancreatic ducts, according to Cambridge Classification (Axon *et al*, 1984) on computed tomography scan and endoscopic retrograde pancreatography, respectively. Etiology of chronic pancreatitis was alcoholic in 30 patients and idiopathic in one patient. No case of pancreatic cancer occurred during the 36-month follow-up in these 31 patients.

### DNA extraction and quantification

Peripheral venous blood samples were collected after informed consent in patients and controls. Blood samples were centrifuged, serum was removed and stored at −20°C until use. DNA was extracted from serum by using the QIAmp Blood Kit (Qiagen, Courtaboeuf, France) according to the blood and body fluid protocol recommended by the manufacturer. Two millilitres of serum were used, and a DNA elution volume of 50 μl was obtained by extraction. The DNA elution was then concentrated to a final volume of 15 μl.

Quantification of serum DNA was performed for all samples using a volume of 3 μl of DNA elution obtained after Qiagen extraction by fluorescence emission after intercalation of Hœschst dye. The fluorescence was read by DyNA Quantô 200 fluorimeter, using the cuve and capillary DyDNA Capillary Cuvette Adaptor Kit (Pharmacia Biotech, Orsay, France). The threshold of DNA detection established by the manufacturer is 2 ng μl^−1^, which corresponds to 100 ng ml^−1^ serum in our extraction protocol (Coulet *et al*, 2000).

### Detection of *KRAS2* gene mutations

Only G12D mutations in codon 12 of the *KRAS2* gene were searched for using allele-specific amplification, with the following primers: 5′-CTTGTGGTAGTTGGAGCTAA-3′, 5′-AATGGTCCTGCACCAGTAATATG-3′. Amplifications were performed with 0.3 μM of each primers, 200 μM of each deoxynucleotide triphosphate (dNTP), 1.5 mM of MgCl_2_, 0.025 units per μl of AmpliTaq Gold polymerase Cetus (Perkin Elmer), 2.5 μl or 5 μl of 10× buffer, 5 μl of the concentrated DNA elution was used as template in a 50 μl volume reaction. Polymerase chain reaction (PCR) with serum DNA was performed 10 min at 94°C, followed by 60 cycles of 94°C for 30 s, 61°C for 30 s, 72°C for 1 min and a final extension of 10 min at 72°C. Controls without DNA and positive controls were performed for each set of PCR reactions. PCR products were separated by electrophoresis in a 6% acrylamide gel and stained with ethidium bromide.

### Serum Ca 19.9 dosage

The serum value of Ca 19.9 was measured with a commercial solid-phase double-antibody sandwich immunoassay (Roche Laboratories, Basel, Switzerland). The upper limit of normal value was 37 UI ml^−1^. All patients and controls also underwent biochemical liver tests. Cholestasis was defined by alkaline phosphatase levels above twice the normal value.

### Statistical analysis

For each marker (serum *KRAS2* mutations and Ca 19.9), sensitivity, specificity, positive and negative predictive values were calculated. Thereafter, the combination of both markers (i.e., one and/or the other positive) was studied. The chi-squared test was used to compare the occurrence of *KRAS2* mutations. Differences were considered significant when *P*<0.05.

## RESULTS

### Circulating DNA quantification

Adequate DNA was extracted from the serum in sufficient quantities for analysis in all patients and controls. All patients but one had a serum DNA concentration higher than the threshold of detection of 100 ng ml^−1^. The mean concentrations of DNA extracted from serum of patients with pancreatic cancer and chronic pancreatitis were 730±90 ng ml^−1^ and 560±93 ng ml^−1^, respectively (*P*=0.19).

### *KRAS2* mutations in circulating DNA

*KRAS2* mutations were identified in the serum of 22 patients (47%) with pancreatic adenocarcinoma and in four patients (13%) with chronic pancreatitis (*P*<0.002). The sensitivity, specificity, positive and negative predictive values of serum *KRAS2* mutations for the diagnosis of pancreatic cancer were 47, 87, 85 and 52%, respectively. There were no statistically significant differences in age, gender, smoking, tumour stage and survival, according to presence or absence of plasma *KRAS2* mutations.

Among patients with chronic pancreatitis, no cancer occurred after a mean follow-up of 36 months (range 13–64), even in those with positive *KRAS2* mutations (with follow-up of 40 months, range 20–61), assessed by clinical observation and abdominal computed tomography scan.

### Serum Ca 19.9 levels

The sensitivity, specificity, positive and negative predictive values of abnormal Ca 19.9 levels for the diagnosis of pancreatic cancer were 91, 87, 91 and 87%, respectively (Table 1

**Table 1:**
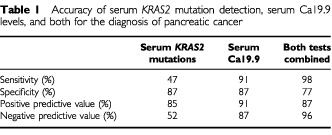
Accuracy of serum *KRAS2* mutation detection, serum Ca19.9 levels, and both for the diagnosis of pancreatic cancer

). Serum Ca 19.9 levels were normal or non interpretable due to cholestasis in 26 patients. Among them, 14 (54%) patients had serum *KRAS2* mutations. Combination of both tests increased sensitivity to 98% with a negative predictive value of 96% for the diagnosis of pancreatic cancer (Table 1).

## DISCUSSION

In the present study, only the most frequent *KRAS2* gene mutation G12D (aspartic acid) observed in pancreatic cancer (Iguchi *et al*, 1996; Tada *et al*, 1998; Castells *et al*, 1999; Watanabe *et al*, 1999) was analysed in the serum of patients and controls, in order to limit the cost of such test and to validate it in clinical practice.

Our study underlines the high feasibility of *KRAS2* mutations analysis, as circulating DNA was obtained in sufficient quantities in all patients. Mean serum DNA concentration were 730 ng ml^−1^, which is comparable to that observed in the series of Mulcahy *et al* (1998).

In the present study, detection of *KRAS2* mutations in circulating DNA had a low sensitivity but a high specificity for the diagnosis of pancreatic cancer. The sensitivity (47%) was in agreement with previous studies (27 to 81%) (Mulcahy *et al*, 1998; Yamada *et al*, 1998; Castells *et al*, 1999; Porta *et al*, 1999; Theodor *et al*, 2000; Zambon *et al*, 2000), although the search for five other possible *KRAS2* mutations in codon 12 was not performed. Higher *KRAS2* mutation prevalences have been reported in pancreatic or duodenal juice (63 to 87%), due probably to higher DNA tumour content in pancreatic juice as compared to plasma (Wilentz *et al*, 1998; van Laethem *et al*, 1998; Watanabe *et al*, 1999). Use of samples of pancreatic juice however requires invasive procedures (fine-needle aspiration during endoscopic ultrasonography or endoscopic retrograde pancreatography). Three studies have reported a higher specificity of serum *KRAS2* mutations compared to the current study (100 *vs* 87%), but their control groups included few patients and essentially healthy subjects (Mulcahy *et al*, 1998; Porta *et al*, 1999; Theodor *et al*, 2000). Since *KRAS2* mutations have been reported in pancreatic tissue or juice from 6–42% of patients with chronic pancreatitis (Furuya *et al*, 1997; Mulligan *et al*, 1999; Lüttges *et al*, 2000; Ha *et al*, 2001), and knowing that a part of this mutated DNA can be released into circulation (Yamada *et al*, 1998), the control group should include patients with chronic pancreatitis. In the series published by Castells *et al* (1999) with the largest control group (including patients with chronic pancreatitis), specificity was comparable (94%) to the present study, and increased in the presence of a pancreatic mass. The accuracy of serum *KRAS2* mutation detection in the differential diagnosis between pancreatic cancer and chronic pancreatitis may be improved by performing quantitative PCR measurement of mutated DNA, in order to discriminate patients with unspecific low levels of mutated DNA (as supposed in chronic pancreatitis) from patients with high levels (pancreatic cancer), as suggested in pancreatic juice analysis by Tada *et al* (1998).

Serum Ca 19.9 is widely used for pancreatic cancer diagnosis with a sensitivity of 80% (Satake and Takeuchi, 1994). This marker is not informative in 5% of population who cannot express serum Ca 19.9 due to Lewis a negative status (Narimatsu *et al*, 1996). However, serum Ca 19.9 lacks specificity (70 to 80%): it can be increased in cholestasis, diabetes mellitus or chronic pancreatitis (Nouts *et al*, 1998). In the present study, serum Ca 19.9 had very good specificity (87%), similar to that of serum *KRAS2* mutations. Combination of both tests could be useful to assess cancer diagnosis in patients with normal or non contributive Ca 19.9 due to cholestasis or negative Lewis a antigen status, and to exclude cancer diagnosis when both tests are negative (predictive negative value of 96%).

In the present study, the presence of *KRAS2* mutations in serum was not correlated to age, gender and smoking habit. It was neither correlated to tumour stage since mutations were detected in plasma of patients with non metastatic tumours, which supports the hypothesis that *KRAS2* mutations are early events in pancreatic carcinogenesis (Jimenez *et al*, 1999). Two studies in the literature are in agreement with this result (Yamada *et al*, 1998; Theodor *et al*, 2000), but another one found a statistically significant relation between circulating DNA *KRAS2* mutations and poor prognosis (Castells *et al*, 1999). Yamada *et al* (1998) have reported disappearance of detectable mutation in plasma after tumoural resection or radio-chemotherapy in six of nine patients, suggesting *KRAS2* mutations may be used as a tumour relapse marker.

Screening for malignancy in patients with chronic pancreatitis is a difficult challenge. Patients with chronic pancreatitis have an increased risk of pancreatic cancer, estimated at 1.8% at 10 years and 4% at 20 years (Lowenfels *et al*, 1993). Three studies have evaluated occurrence of pancreatic cancer in patients with chronic pancreatitis with respect to *KRAS2* mutations in pancreatic juice: only one found an increase in pancreatic cancer in patients with *KRAS2* mutations with methodological limitations (few cases, early diagnosis of cancer after inclusion) (Furuya *et al*, 1997; Lohr *et al*, 2001; Queneau *et al*, 2001). To our knowledge, no study focused on the incidence of pancreatic cancer in patients with chronic pancreatitis according to serum *KRAS2* mutations. In the present study, follow-up was too short to demonstrate an increased risk of cancer in patients with chronic pancreatitis and serum *KRAS2* mutations.

In conclusion, although detection of plasma *KRAS2* mutations in circulating DNA is not a definitive argument for malignancy, it could contribute to cancer diagnosis. This test seems particularly interesting in patients with normal or inconclusive Ca 19.9 levels due to cholestasis or Lewis a negative status. In patients with normal serum Ca 19.9 levels and no *KRAS2* mutation, the diagnosis of pancreatic cancer can be excluded with almost certainty.
